# Preclinical Testing of a Vaccine Candidate against Tularemia

**DOI:** 10.1371/journal.pone.0124326

**Published:** 2015-04-21

**Authors:** Ragavan Varadharajan Suresh, Zhuo Ma, Raju Sunagar, Vivek Bhatty, Sukalyani Banik, Sally V. Catlett, Edmund J. Gosselin, Meenakshi Malik, Chandra Shekhar Bakshi

**Affiliations:** 1 Department of Microbiology and Immunology, New York Medical College, Valhalla, United States of America; 2 Albany College of Pharmacy and Health Sciences, Albany, United States of America; 3 Center for Immunology and Microbial Disease, Albany Medical College, Albany, United States of America; Midwestern University, UNITED STATES

## Abstract

Tularemia is caused by a gram-negative, intracellular bacterial pathogen, *Francisella tularensis (Ft)*. The history weaponization of *Ft* in the past has elevated concerns that it could be used as a bioweapon or an agent of bioterrorism. Since the discovery of *Ft*, three broad approaches adopted for tularemia vaccine development have included inactivated, live attenuated, or subunit vaccines. Shortcomings in each of these approaches have hampered the development of a suitable vaccine for prevention of tularemia. Recently, we reported an oxidant sensitive mutant of *Ft* LVS in putative EmrA1 (FTL_0687) secretion protein. The *emrA1* mutant is highly sensitive to oxidants, attenuated for intramacrophage growth and virulence in mice. We reported that EmrA1 contributes to oxidant resistance by affecting the secretion of antioxidant enzymes SodB and KatG. This study investigated the vaccine potential of the *emrA1* mutant in prevention of respiratory tularemia caused by *Ft* LVS and the virulent SchuS4 strain in C57BL/6 mice. We report that *emrA1* mutant is safe and can be used at an intranasal (i. n.) immunization dose as high as 1x10^6^ CFU without causing any adverse effects in immunized mice. The *emrA1* mutant is cleared by vaccinated mice by day 14–21 post-immunization, induces minimal histopathological lesions in lungs, liver and spleen and a strong humoral immune response. The *emrA1* mutant vaccinated mice are protected against 1000–10,000LD_100_ doses of i.n. *Ft* LVS challenge. Such a high degree of protection has not been reported earlier against respiratory challenge with *Ft* LVS using a single immunization dose with an attenuated mutant generated on *Ft* LVS background. The *emrA1* mutant also provides partial protection against i.n. challenge with virulent *Ft* SchuS4 strain in vaccinated C57BL/6 mice. Collectively, our results further support the notion that antioxidants of *Ft* may serve as potential targets for development of effective vaccines for prevention of tularemia.

## Introduction

Tularemia is a disease caused by a gram-negative, intracellular bacterial pathogen *Francisella tularensis* (*Ft*). The history of *Ft* weaponization has been documented by Japan, the former Soviet Union, and the United States [1,2]. This history has generated concerns regarding the potential use of *Ft* as a bioweapon or as an agent of bioterrorism [1,3,4,5]. Nonspecific symptoms of tularemia and the engineered antibiotic resistant strains undermine therapeutic options. In the last hundred years since the discovery of *Ft*, three broad approaches including inactivated, live attenuated, or subunit vaccines have been employed for tularemia vaccine development, but none of them have been successful [6,7,8,9,10,11,12,13]. Although, a Live Vaccine Strain (LVS) developed from the Russian strain *Ft* subspecies *holarctica* S15 is protective, it retains residual virulence in humans when immunized via aerosol or intranasal (i.n.) routes. *Ft* LVS is not approved by the US Food and Drug Administration for mass immunizations in the USA due to adverse reactions and residual virulence. Live attenuated mutants containing single gene deletions of the highly virulent category A Select Agent *Ft* SchuS4 strain have shown better protective efficacy than the *Ft* LVS [14]. However, the possibility of reversion of these mutants to fully virulent form is hampering their development into effective vaccines. Double or multiple gene deletion mutants of both the *Ft* LVS and SchuS4 strains which may not revert to virulent form are hyper-attenuated and fail to render any protection against i.n. challenge with *Ft* SchuS4. Inactivated *Ft* LVS or SchuS4 vaccines do not protect against virulent *Ft* [8,15,16] and subunit vaccines have been shown to possess limited protective ability due to the lack of a suitable platform for delivering multiple protective antigens simultaneously [7]. Collectively, these shortcomings have hampered the development of a suitable vaccine for prevention of tularemia.

Our previous work has demonstrated that the antioxidant mutant of *Ft* LVS carrying a point mutation in iron-containing superoxide dismutase gene (*sodB*) is partially attenuated for virulence in mice [17]. The *sodB* mutant when used as a vaccine protected 100% of BALB/c (unpublished data) and 40% of C57BL/6 mice against a lethal i.n. challenge with virulent *Ft* SchuS4 strain. The loss of SodB results in upregulation of several immunogenic proteins in the *sodB* mutant of *Ft* LVS [13]. Further, the catalase (KatG) of *Ft* LVS has been implicated in the suppression of host immune responses and evidence suggests that this antioxidant enzyme of *Ft* inhibits redox-sensitive signaling components to suppress innate immune responses of the host [18]. These findings indicate that antioxidant defenses of *Ft* LVS, specifically SodB and KatG may serve as potential targets for further vaccine development. Our observations are further supported by studies conducted using a modified BCG vaccine [19]. It has been reported that the BCG strain with diminished antioxidants was safer, persisted less than the parent BCG following vaccination, and provided greater protection against aerosolized challenge with *Mycobacterium tuberculosis*. The modified BCG deficient in antioxidant defenses when used as a vaccine, induces stronger immune response and enhanced recall response upon subsequent challenge with virulent *M*. *tuberculosis* [19].

Recently, we reported an oxidant-sensitive mutant of *Ft* LVS in putative EmrA1 (FTL_0687) secretion protein. We observed that the *emrA1* mutant is highly sensitive to oxidants, and is attenuated for intramacrophage growth and virulence in mice [20]. Further investigations revealed that EmrA1 contributes to oxidant resistance by affecting secretion of antioxidant enzymes SodB and KatG. Further characterization of the *emrA1* mutant revealed phenotypes characteristics of both the *sodB* and Δ*katG* mutants of *Ft* LVS [20]. Based on the success of the *sodB* vaccine and the potential of KatG in the modulation of host’s immune response, in this study we investigated the vaccine potential of the *emrA1* mutant of *Ft* LVS in prevention of experimental respiratory tularemia in C57BL/6 mice. We report that the *emrA1* mutant is safe and can be used i.n. at an immunization dose as high as 1x10^6^ CFU without causing any adverse effects in immunized mice, is cleared by the vaccinated mice by day 14–21 post-immunization, induces minimal histopathology in lungs, liver and spleen, elicits a strong humoral immune response, and protects against 1000–10,000LD_100_ doses of i.n. *Ft* LVS challenge. The *emrA1* mutant also provides partial protection against i.n. challenge with virulent *Ft* SchuS4 strain in vaccinated C57BL/6 mice. Collectively, these findings further support the notion that antioxidants of *Ft* may serve as potential targets for development of effective vaccines for prevention of tularemia.

## Materials and Methods

### Ethics Statement

This study was carried out in strict accordance with the recommendations and guidelines of National Council for Research (NCR) for care and use of animals. All the animal experiments were conducted in the centralized Animal Resources Facilities of Albany Medical College and New York Medical College licensed by the USDA and the NYS Department of Health, Division of Laboratories and Research and accredited by the American Association for the Accreditation of Laboratory Care. The use of animals and protocols were approved by the Institutional Animal Care and Use Committee (IACUC) of Albany Medical College (Protocol Number 12–01001) and New York Medical College (Protocol Number 63-2-1213H). Mice were administered an anesthetic cocktail consisting of ketamine (5 mg/kg) and xylazine (4 mg/kg) and underwent experimental manipulation only after they failed to exhibit a toe pinch reflex. Mice exhibiting more than 20% weight loss, anorexia, dehydration and impairment of mobility were removed from the study and euthanized by approved means. Humane endpoints were also necessary for mice which survived at the conclusion of the experiment. Mice were administered an anesthetic cocktail of ketamine and xylazine intraperitoneally and then euthanized via cervical dislocation followed by cardiac puncture, a method that is consistent with Albany Medical College GLP Standard Operating Procedure on “Guidelines for Animal Euthanasia” (Document Number ARF-VC-004) and recommendations of the Panel on Euthanasia of the American Veterinary Medical Association. In all experimental procedures, efforts were made to minimize pain and suffering.

### Bacterial Strains

*Ft* LVS was obtained from BEI Resources (Manassas, VA). *Ft* SchuS4 used for challenge experiments was obtained from the U.S. Army Medical Research Institute for Infectious Diseases (USAMRIID, Frederick, MD). The *emrA1* mutant (*FTL_0687*) as reported earlier was identified by screening transposon mutants of *Ft* LVS in the presence of H_2_O_2_ [20]_._ All the *Francisella* strains were cultured in Mueller Hinton Broth (MHB) or MH-chocolate agar plates as described previously [21,22]. All experiments involving *Ft* SchuS4 were conducted in CDC approved BSL3/Animal BSL3 facility of Albany Medical College, NY.

### Mice

C57BL/6 mice (Charles River laboratories, NY) used in this study were maintained in a specific pathogen free environment in the Animal Facility of New York Medical College or Albany Medical College. All mice used in the experiment were six to eight weeks old of either sex. Prior to intranasal inoculations or experimental manipulations, mice were anesthetized via intraperitoneal injection of ketamine/xylazine cocktail. In all experimental procedures, efforts were made to minimize pain and suffering. All immunization and challenge experiments with *Ft* LVS were conducted in the Animal Facility of New York Medical College; while those with *Ft* SchuS4 were conducted in the Animal BSL3 facility of Albany Medical College. All the animal experiments were conducted according to the protocols approved by the IACUC at New York Medical College and Albany Medical College.

### Immunizations

Mice were given a single i.n. immunization dose of ~1x10^6^CFU of the *emrA1* mutant in a 20μL (10 μL/nostril) suspension per mouse. The immunized mice were observed daily for clinical signs, morbidity, mortality, and weight loss. The immunized mice were challenged on day 42 post-primary immunizations. In experiments testing safety of the *emrA1* mutant vaccine, equal numbers of C57BL/6 mice were inoculated i.n. with 1x10^6^ CFU of *Ft* LVS as controls.

For determination of the protective efficacy of the *emrA1* mutant vaccine against the *Ft* SchuS4 challenge, various vaccination regimens were adopted. Mice were either immunized with 1x10^6^ CFU of the *emrA1* i.n. as a single dose; or an additional booster was given on day 21 of the primary immunization. Additionally, mice were first immunized i.n. and then boosted via intradermal (i.d.) route with 1x10^6^ CFU of the *emrA1* mutant on day 0 and day 21 respectively; or were first immunized via the i.d. route followed by a booster vaccination given by i.n. route. Based on observations that killed *Ft* LVS complexed with anti-*Ft* LPS monoclonal antibodies provides better protection against *Ft* SchuS4 than killed *Ft* LVS alone, we complexed 1x10^6^ CFU of the *emrA1* mutant with 5μg of anti-*Ft* LPS monoclonal antibodies (*emrA1-*mAb) following the protocol described earlier [23]. Mice were immunized either with a single dose of *emrA1-*mAb or boosted on day 21; or immunized alternatively using i.n. priming followed by i.d. booster on day 21 and vice-versa. Finally, mice were also immunized with a low dose of 1x10^3^ CFU of the *emrA1* mutant and boosted with this low dose on day 21 of the primary immunization using alternate i.n. or i.d. inoculations as described above before being challenged i.n. with *Ft* SchuS4.

### Challenge

Mice were challenged i.n. with 1x10^7^ or 1x10^8^ CFU of *Ft* LVS after 42 days post-primary immunization. To determine the duration of immunity rendered by the *emrA1* vaccine, the immunized mice were also challenged i.n. with 1x10^7^ CFU of *Ft* LVS day 75 post-primary immunization. The protective efficacy of the *emrA1* mutant vaccine against *Ft* SchuS4 was determined by challenging immunized mice with *Ft* SchuS4 on day 42 post-primary immunization. Mice were observed for morbidity and mortality for a period of 21 days. The survival data is represented as Kaplan-Meier survival curves and the statistical significance was determined by Log-Rank test. The immunization and challenge schedules are shown in **Table 1**.

**Table 1:** Immunization and challenge schedules used in this study.

Vaccine	Primary Immunization		Booster Immunization**		Challenge Strain	Challenge***		Time of challenge (Days)****
	Dose (CFU)	Route	Dose	Route		Dose (CFU)	Route	
***emrA1* Mutant**	1x10^6^	i.n.	-	-	*Ft* LVS	1x10^7^	i.n.	42
	1x10^6^	i.n.	-	-	*Ft* LVS	1x10^8^	i.n.	42
	1x10^6^	i.n.	-	-	*Ft* LVS	1x10^7^	i.n.	75
	1x10^6^	i.n.	-	-	*Ft* SchuS4^#^	32	i.n.	21
	1x10^6^	i.n.	1x10^6^	i.n.	*Ft* SchuS4	38	i.n.	42
	1x10^6^	i.n.	1x10^6^	i.d.	*Ft* SchuS4	17	i.n.	42
	1x10^6^	i.d.	1x10^6^	i.n.	*Ft* SchuS4	17	i.n.	42
	1x10^3^	i.n.	1x10^3^	i.d.	*Ft* SchuS4	23	i.n.	42
	1x10^3^	i.d.	1x10^3^	i.n.	*Ft* SchuS4	24	i.n.	42
*emrA1* Mutant-mAb complex*	1x10^6^	i.n.	-	-	*Ft* SchuS4	32	i.n.	21
	1x10^6^	i.n.	1x10^6^	i.d.	*Ft* SchuS4	17	i.n.	42
	1x10^6^	i.d.	1x10^6^	i.n.	*Ft* SchuS4	17	i.n.	42

*emrA1 mutant was complexed with 5μg/ml of anti-*Ft* LVS LPS monoclonal antibodies

**Booster vaccinations were administered on day 21 of the primary immunization

***Equal doses of *Ft* LVS or SchuS4 were administered i.n. to unvaccinated control mice

****Days post-primary immunization

^#^ The target dose for all *Ft* SchuS4 challenge experiments was 25 CFU. The actual CFU administered are shown.

i.n. = Intranasal; i.d. = Intradermal; mAb = Monoclonal antibodies.

### Post-immunization and post-challenge studies

#### Kinetics of bacterial clearance and histopathology

All mice following vaccination and challenge were weighed periodically to determine the progression of disease. Mice were bled by retro-orbital venipuncture to collect serum for determination of *Ft* specific antibodies. Mice were sacrificed on days 1, 5, 7, 14 and 21 post-immunization; and on days 5, 7 and 14 following challenge with *Ft* LVS. Lungs of the immunized and challenged mice were inflated with sterile PBS and excised aseptically. The liver and spleen were also removed aseptically. The right lobe of the lung and small portions of liver and spleen were stored in 10% formalin for histopathological evaluations. Lung, liver and spleen homogenates were prepared in a bead- beater using sterile zirconia beads, diluted ten-fold, and plated on chocolate agar plates for quantitation of bacterial numbers as described earlier [13]. The remaining lung and spleen homogenates were spun at 10,000 rpm for 10 minutes to remove the tissue debris. The clear supernatants were collected and used for quantitation of pro-inflammatory cytokines in lung and spleen. For histopathological evaluation, the organs were paraffin embedded, sectioned and stained with hematoxylene and eosin (H&E). The H&E stained sections were observed under light microscope and images were taken.

#### Pro-inflammatory cytokine analysis

Levels of pro-inflammatory cytokines tumor necrosis factor-alpha (TNF-α), interleukin-6 (IL-6), macrophage chemotactic protein (MCP-1), interleukin-12 (IL-12), interferon-gamma (IFN-γ) and interleukin-10 (IL-10) were measured in lung and spleen homogenates. Mouse inflammation kit (BD Biosciences) was used for the simultaneous quantitation of these pro-inflammatory cytokines as previously described [13].

#### Quantitation of *Ft* specific antibodies

*Ft* specific total IgG, IgG2a, IgG2b, IgG1 and IgA levels were quantitated in sera samples collected from immunized and challenged mice at various time intervals by ELISA. The plates were coated with *Ft* LVS lysates and the ELISA was performed using standard procedure.

### Statistical analysis

All results were expressed as Means ± S.D or Mean ± SEM. Statistical comparisons between the groups were made using one-way ANOVA followed by Tukey-Kramer multiple comparison test. Survival results were plotted as Kaplan-Meier survival curves. Differences between the experimental groups were considered statistically significant at a *P* < 0.05 level. The data were statistically analyzed using GraphPad and InStat software.

## Results

### Mice immunized with the *emrA1* mutant initially lose their body weight but rapidly regain it back

**Fig 1:**
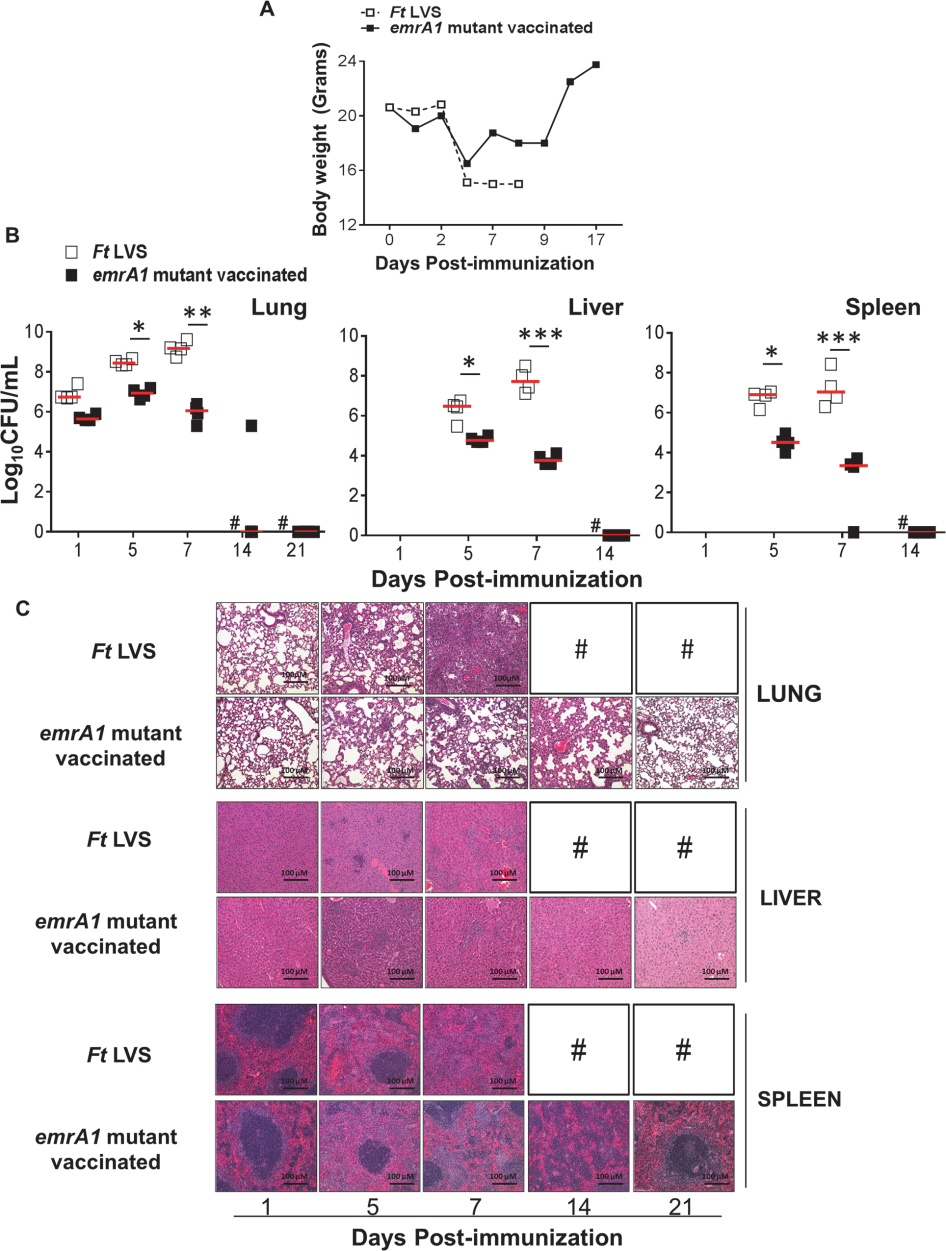
Immunization with the *emrA1* mutant results in minimal weight loss, rapid bacterial clearance, and histopathological lesions in lung, liver and spleen. C57BL/6 mice were immunized i.n. with 1×10^6^ CFU of the *emrA1* mutant. Mice infected with equal numbers of wild type *Ft* LVS were used as controls. **(A)** The immunized mice were weighed at the indicated times post-immunization to track the progress of infection. **(B)** On days 1, 5, 7, 14 and 21 post-immunization, mice (n = 4 per group/time point) were euthanized and bacterial burdens were quantified in their lung, liver and spleen. Bacterial counts in organs are expressed as Log_10_CFU/mL. The *P* values were determined using one way ANOVA. *P<0.05; **P<0.01; ***P<0.*001*. **(C)** Excised lungs, livers and spleens were preserved in 10% formalin, paraffin embedded, sliced into 5 μM thin sections and stained with Hematoxylene & Eosin. Stained sections were observed for histopathological lesions under a light microscope (Magnification 100×). # = *Ft* LVS infected mice succumbed to infection.

We have recently reported that the *emrA1*mutant of *Ft* LVS is highly attenuated for virulence and doses as high as 1x10^5^ or 1x10^6^ CFU administered i.n. do not cause any mortality in infected mice [20]. To determine the vaccine potential of the *emrA1* mutant, we first investigated the safety of this mutant strain in C57BL/6 mice. Mice were immunized i.n. with 1x10^6^ CFU of the *emrA1* mutant and observed for any morbidity by determining the body weight, kinetics of bacterial clearance, and histopathological changes observed in lungs, liver and spleen. Mice infected with a similar dose of wild type *Ft* LVS were included as controls. It was observed that similar to mice infected with *Ft* LVS, those immunized with the *emrA1* mutant did not show any changes in body weight for first three days, which was followed by a sudden weight loss by day 5 post-immunization. However, the *emrA1* mutant immunized mice started to regain their body weights by day 7 and were over their initial body weights by day 17 post-immunization. Contrary to this, the *Ft* LVS infected mice did not regain their weights after day 5 post-infection and 100% mice succumbed to infection by day 8 post-infection (**Fig 1A**).

### Mice immunized with the *emrA1* mutant clear bacteria by day 14–21 post-immunization

We determined the kinetics of bacterial clearance in mice immunized with 1x10^6^ CFU of the *emrA1* mutant by quantitating the bacterial load in lung, liver and spleen on days 1, 5, 7, 14 and 21 post-immunization. It was observed that the numbers of *emrA1* mutant went up nearly fivefold by day 5 as compared to day 1 post-immunization and then gradually reduced and were completely cleared from the lungs by day 21 post-immunization. Contrary to this, the bacterial numbers went up exponentially in the lungs of mice infected with 1x10^6^ CFU of *Ft* LVS and peaked at day 7 post-infection following which 100% of these mice died. The *emrA1* mutant disseminated to liver and spleen; however, significantly lower bacterial numbers were observed on days 5 and 7 post-immunization as compared to those observed in *Ft* LVS infected mice. The *emrA1* mutant bacteria were completely cleared from liver and spleen by day 14 post-infection whereas in *Ft* LVS infected mice, the bacterial numbers increased from days 5 to 7 post-infection (**Fig 1B**). These results indicate that the *emrA1* mutant is cleared by mice despite a high immunization dose by day 14 from liver and spleen, and by day 21 from lungs. These results also indicate that the *emrA1* mutant, although attenuated for virulence, survives in host for a length of time that may be sufficient to induce an effective immune response [21].

### Mice immunized with the *emrA1* mutant induce minimal histopathological lesions in lung, liver and spleen

We next examined the histopathological lesions in lung, liver, and spleen of mice immunized with 1x10^6^ CFU of the *emrA1* mutant and compared with those observed in mice infected with a similar dose of *Ft* LVS. The lesions in the lungs of *Ft* LVS infected mice progressed from patchy inflammatory foci to severe inflammation (day 5 post-infection), consolidation of lungs, and necrotizing pneumonia (day 7 post-infection). However, no such lesions were observed in the lungs of the *emrA1* mutant immunized mice and lung sections appeared normal except for focal inflammatory lesions on day 5 and 7 post-infection which were resolved completely by day 21 post-immunization. The livers of *Ft* LVS mice revealed progressive development of granulomas from day 5 to day 7 post-infection which eventually collapsed and became necrotic by day 7 post-infection. Granulomas were also observed in livers from the *emrA1* mutant immunized mice on day 5 post-immunization. However, these granulomas started to resolve by day 7 and were not observed by day 14–21 post-immunization. Similar to lung and liver, severe histopathological lesions were also observed in the spleen of *Ft* LVS infected mice. These lesions were far less severe in the emrA1 mutant immunized mice and were associated with proliferation of germinal centers by day 7 and started to resolve by day 14 and reverted back to normal architecture by day 21 post-immunization (**Fig 1C**).

Collectively, these results demonstrate that mice can tolerate 1x10^6^ immunization dose of the *emrA1* mutant and quickly regain their body weights. The *emrA1* mutant is cleared by day 14–21 post-immunization without causing extensive pathological changes in lung, liver and spleen indicating that this mutant is safe as a vaccine.

### Mice immunized with the *emrA1* mutant induce regulated production of pro-inflammatory cytokines and a potent antibody response

**Fig 2:**
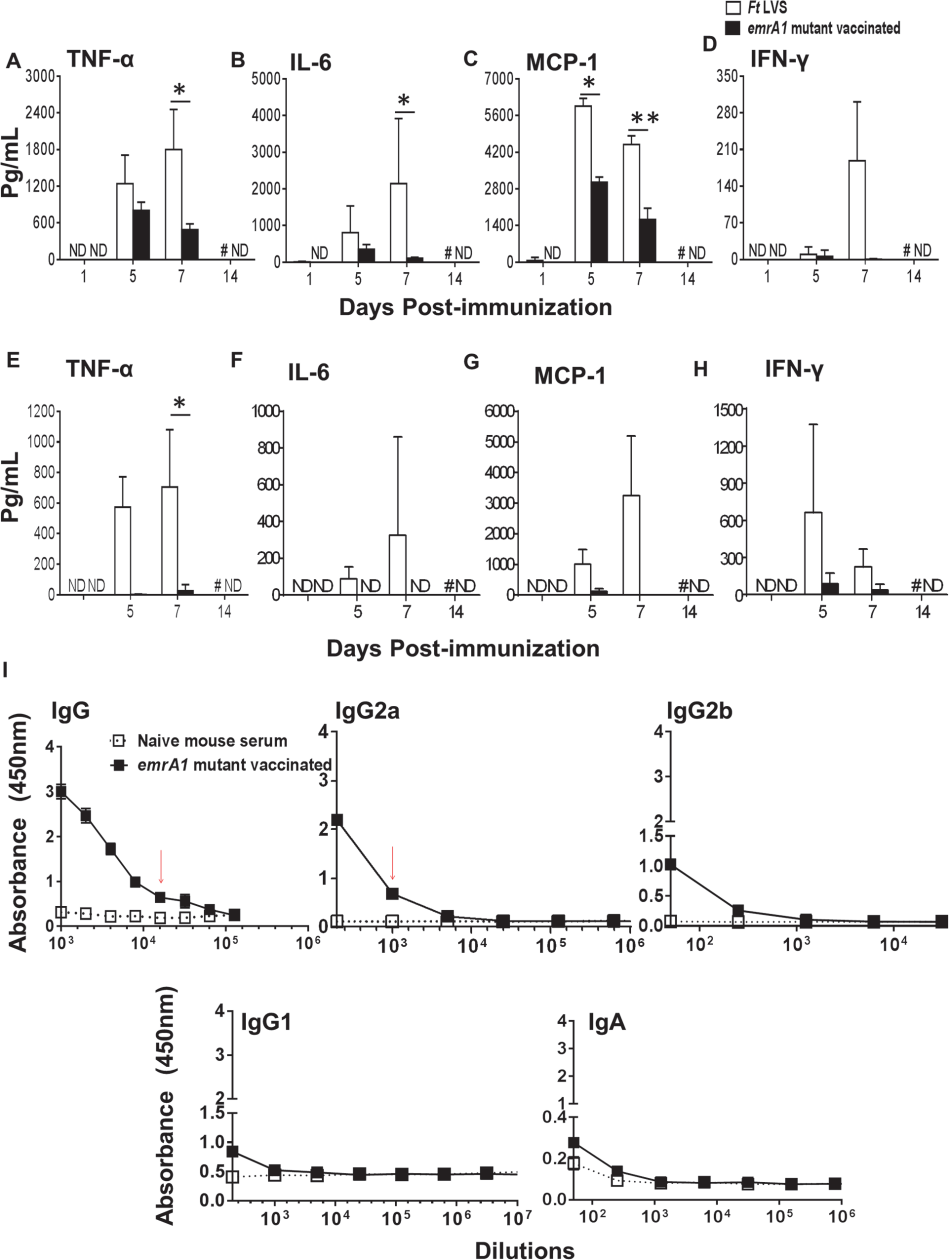
Mice immunized with the *emrA1* mutant induce regulated production of pro-inflammatory cytokines and a potent antibody response. C57BL/6 mice were immunized i.n. with *1*×10^6^ CFU of the *emrA1* mutant or *Ft* LVS. On days 1, 5, 7 and 14 post-immunization, mice (n = 4 per group/time point) were euthanized and their excised lungs and spleens were homogenized. Clear lung **(A-D)** and spleen **(E-H)** homogenates were used for quantification of indicated pro-inflammatory cytokines using flow cytometric analysis. The data are represented as Mean ± S.D. The *P* values were determined using one way ANOVA. *P<0.05; **P<0.*01*. **(I)** On day 42 post-immunization, mice (n = 3 per group/ time point) were anesthetized and bled retroorbitally to obtain serum. *Ft* specific total IgG, IgG2a, IgG2b, IgG1 and IgA levels in serum samples were determined by ELISA. The data are represented as Mean ± S.D. of absorbance values measured at 450 nm. Red arrows indicate antibody titers. # = *Ft* LVS immunized mice succumbed to infection; ND = Not detected.

We determined the levels of pro-inflammatory cytokines TNF-α, IL-6, MCP-1, and IFN-γ in mice immunized with 1x10^6^ CFU of the *emrA1* mutant and compared with those observed in mice infected with an equal dose of *Ft* LVS on days 1, 5, 7, and 14 post-immunization. None of the four cytokines were detected on day 1 post-immunization in the lung homogenates of either the *emrA1* mutant or *Ft* LVS infected mice. The levels of all four cytokines increased gradually thereafter and were significantly elevated in *Ft* LVS infected mice on day 7 post-infection. Significantly elevated levels of MCP-1 were also observed in *Ft* LVS infected mice on day 5 post-infection. These results indicate that infection of mice with 1x10^6^ CFU of *Ft* LVS which is 100 times the LD_100_ dose result in a cytokine storm which is usually followed by death. Contrary to this, in the lung homogenates of the *emrA1* mutant immunized mice, the levels of these pro-inflammatory cytokines peaked at day 5 and gradually reduced by day 7 post-immunization before returning to the baseline values (**Fig 2A–2D**). The pattern of pro-inflammatory cytokines in spleens of *Ft* LVS infected mice was similar to that observed in lungs with peak levels observed at day 7 post-infection. However, very low to undetectable levels of TNF-α, IL-6, MCP-1 and IFN-γ were observed in spleens of the *emrA1* mutant immunized mice (**Fig 2E–2H**). Furthermore, no detectable levels of anti-inflammatory cytokine IL-10 were observed in lung or spleen homogenates of mice immunized with the *emrA1* mutant. These results indicate that mice immunized with the *emrA1* mutant induce regulated production of pro-inflammatory cytokines which may be associated with reducing bacterial load (**Fig 1B**). Further, lower levels of these cytokines also inflict less pathology observed in the lungs and spleens of the *emrA1* immunized mice as compared to the *Ft* LVS infected mice (**Fig 1C**).

We also determined the levels of *Ft* LVS specific total IgG, IgG2a, IgG2b, IgG1 and IgA levels in sera collected from the *emrA1* immunized mice on day 42 post-immunization, the time at which the immunized mice were challenged. A potent antibody response comprising of *Ft* specific total IgG and IgG2a antibodies was observed in mice immunized with the *emrA1* mutant. On the other hand, very low levels of IgG2b, IgG1 and IgA antibodies were observed in the immunized mice (**Fig 2I**).

### Mice immunized with the *emrA1* mutant are protected against 1000LD_100–_10,000LD_100_ challenge dose of *Ft* LVS

**Fig 3:**
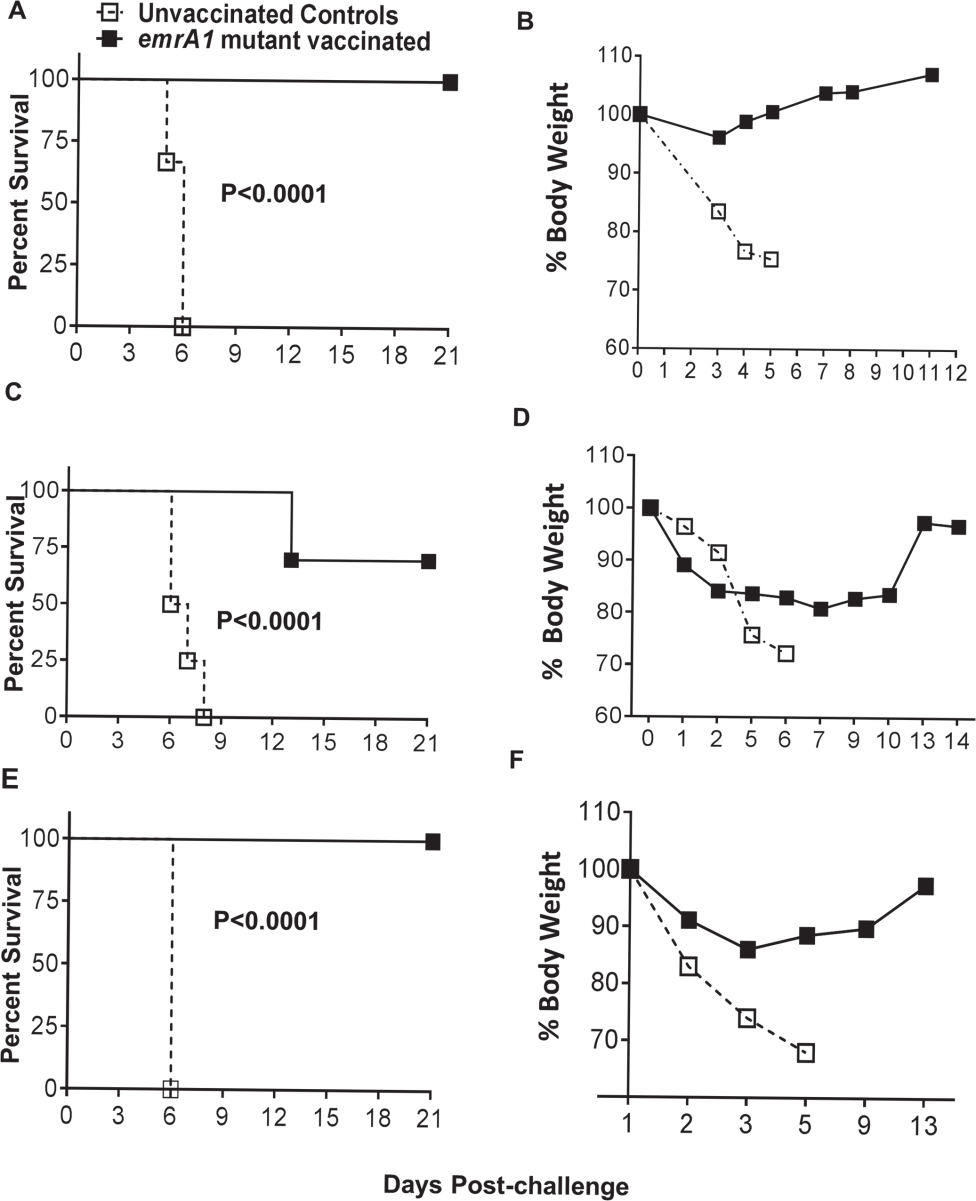
Mice immunized with the *emrA1* mutant are protected against 1000LD100–10,000LD100 challenge dose of *Ft* LVS. C57BL/6 mice (n = 5–10 per group) were immunized i.n. with 1×10^6^ CFU of the *emrA1* mutant. **(A, B)** On day 42 of the primary immunization mice were challenged i.n. with 1×10^7^ CFU of wild type *Ft* LVS. Age matched unvaccinated mice challenged with a similar dose of *Ft* LVS were kept as controls. (**C, D)** On day 42 of the primary immunization mice were challenged i.n. with 1×10^8^ CFU of wild type *Ft* LVS. Age matched unvaccinated mice challenged with a similar dose of *Ft* LVS were kept as controls. (**E, F)** On day 75 of the primary immunization mice were challenged i.n. with 1×10^7^ CFU of wild type *Ft* LVS. Age matched unvaccinated mice challenged with a similar dose of *Ft* LVS were kept as controls. The Challenged mice were observed for morbidity and mortality for a period of 21 days post-challenge (**A, C, E**). The mice were weighed at the indicated times post-challenge to monitor the progression of infection (**B, D, F**). The survival results are expressed as Kaplan-Meier survival curves and P values were determined by Log-rank test. Body weights of mice are expressed percent body weights.

The LD_100_ dose of *Ft* LVS in our hands is 1x10^4^ CFU by the i.n. route. To determine the protective efficacy of the *emrA1* mutant vaccine, we challenged the *emrA1* mutant immunized mice with 1x10^7^ (1000LD_100_) or 1x10^8^ (10,000LD_100_) CFU of wild type *Ft* LVS by i.n. route. It was observed that 100% of C57BL/6 mice immunized with the *emrA1* mutant and challenged with 1x10^7^ CFU of *Ft* LVS survived until day 21 post-challenge at which time the experiment was terminated. All the unvaccinated control mice that received similar challenge dose died by day 6 post-challenge. The *emrA1* mutant immunized mice exhibited minimal loss of body weight following the challenge whereas majority of unvaccinated controls lost more than 20% of their body weight by day 5 post-challenge were removed from the study and euthanized (**Fig 3A and 3B**). When the challenge dose was increased to 1x10^8^ CFU of *Ft* LVS, 70% (7/10) mice immunized with the *emrA1* mutant survived the challenge. The *emrA1* mutant immunized mice receiving the higher challenge dose showed reduction in body weight for first two days post-challenge which remained unchanged till day 10 after which these mice regained their starting body weights. The unvaccinated controls lost their body weights quickly and either succumbed to the challenge dose or removed from the study if the weight loss was more than 20% of their starting body weight (**Fig 3C and 3D**).

To determine the duration of immune protection, mice immunized with 1x10^6^ CFU of the *emrA1* mutant were challenged with 1x10^7^ CFU of *Ft* LVS on day 75 of the primary immunization. One hundred percent of mice that received this challenge dose survived and showed a weight loss pattern similar to that observed for mice challenged with a similar dose on day 42 post-primary immunization (**Fig 3E and 3F**).

Collectively, these data demonstrate that *emrA1* mutant is not only safe but also provides protection against extremely high challenge doses of *Ft* LVS. In addition, the protective immune response is maintained against a 1000LD_100_ challenge dose until day 75 after a single dose immunization indicating a prolonged period of immune protection.

### The *emrA1* mutant vaccinated mice clear bacteria rapidly and exhibit minimal histopathological lesions in lung, liver and spleen following lethal *Ft* LVS challenge

**Fig 4:**
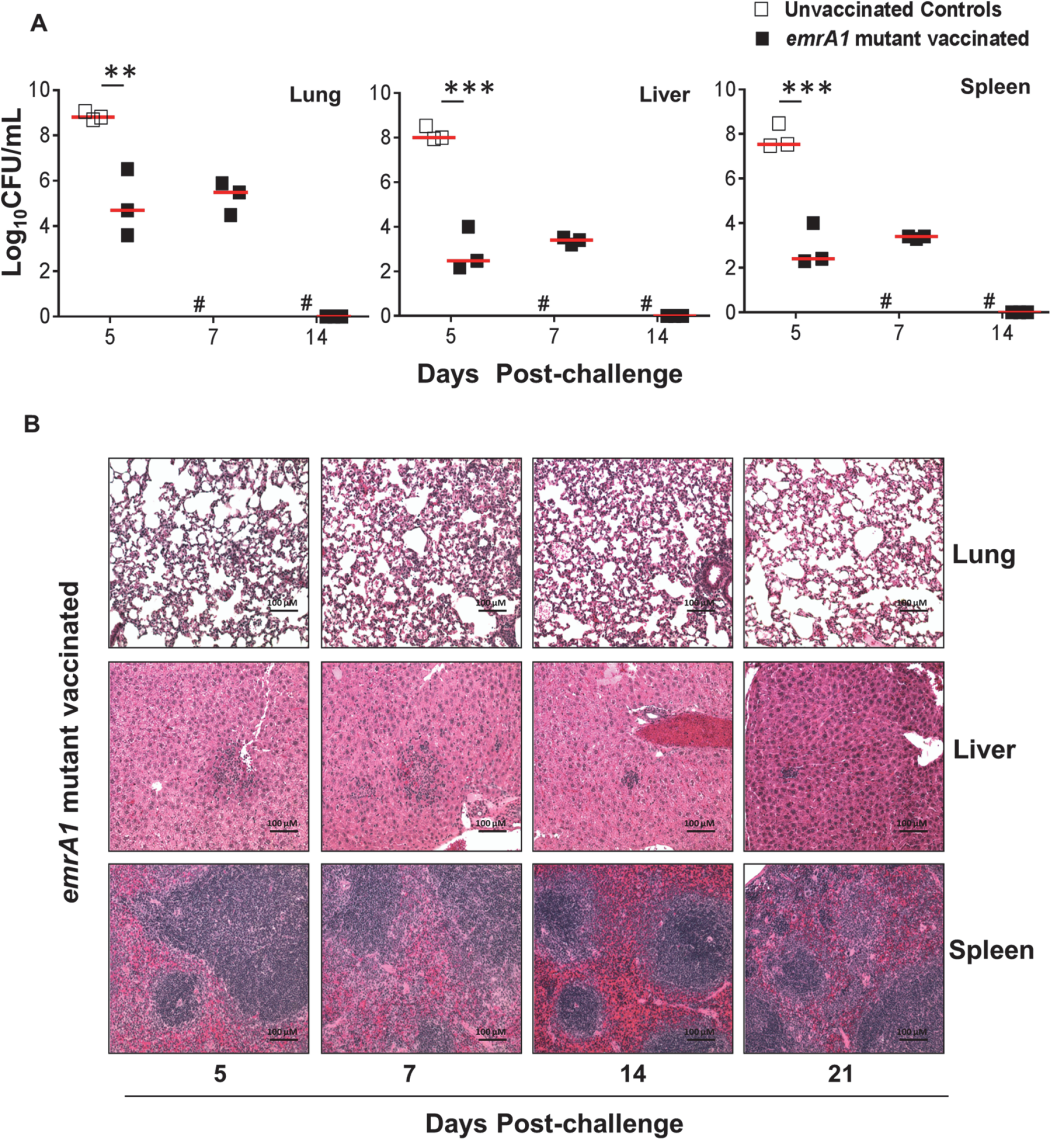
The *emrA1* mutant vaccinated mice clear bacteria rapidly and exhibit minimal histopathological lesions in lung, liver and spleen following lethal *Ft* LVS challenge. C57BL/6 mice immunized i.n. with 1×10^6^ CFU of the *emrA1* mutant or the unvaccinated control mice were challenged i.n. with 1×10^7^ CFU of *Ft* LVS 42 days post-immunization. **(A)** On days 5, 7, and 14 post-challenge, mice (n = 3 per group/time point) were euthanized and bacterial burdens were quantified in their lung, liver and spleen. Bacterial counts in organs are expressed as Log_10_ CFU/mL. The *P* values were determined using one way ANOVA. **P<0.*01;* ***P<0.*001*. **(B)** Lungs, livers and spleens collected at the indicated times post-challenge were preserved in 10% formalin, embedded in paraffin blocks, sliced into 5 μM thin sections and stained with Hematoxylene & Eosin. Stained sections were observed for histopathological lesions under a light microscope (Magnification 100×). # = Unvaccinated mice succumbed to infection.

We observed that 100% of mice immunized with the *emrA1* mutant and challenged i.n. with 1x10^7^ CFU of *Ft* LVS survived the infection. We next investigated the kinetics of bacterial clearance in vaccinated mice on days 5, 7 and 14 following the challenge. The bacterial numbers recovered from lungs of the vaccinated mice were nearly 20-fold lower than the challenge dose on day 5 post-challenge. On the contrary, these numbers were nearly 20-fold higher in the lungs of unvaccinated controls. On day 7 post-challenge, the bacterial numbers in the lungs of *emrA1* mutant vaccinated mice remained similar to those observed for day 5 and were cleared completely and became undetectable by day 14 post-challenge. Similar patterns were also observed for bacterial numbers recovered from livers and spleens of the *emrA1* mutant vaccinated and challenged mice. However, the bacterial numbers recovered from liver and spleens were much lower than those observed in the lungs of the vaccinated mice (**Fig 4A**). These results indicate that the *emrA1* mutant vaccinated mice clear *Ft* very rapidly upon an extremely lethal challenge.

We also evaluated histopathological lesions in lung, liver and spleen of *emrA1* mutant vaccinated mice challenged with 1x10^7^ CFU of *Ft* LVS on days 5, 7, 14 and 21 post-challenge. As observed upon immunization, the *emrA1* mutant vaccinated mice exhibited minimal pathological lesions consisting of mild inflammatory foci in lungs on days 5 and 7 post-challenge. No lesions were observed in *emrA1* vaccinated and *Ft* LVS challenged mice on days 14 and 21 post-challenge. Liver showed formation of granulomas on day 5 post-challenge. These granulomas gradually reduced in size and very small granulomas were observed on day 21 post-challenge even when bacteria were completely cleared from liver. The splenic architecture of the *emrA1* mutant vaccinated mice remained unaltered on day 5 and 7 post-challenge; however, proliferation of germinal centers were observed on days 14 and 21 post-challenge again at a time when bacteria were not recovered from the spleens (**Fig 4B**). Collectively, these results demonstrate that mice immunized with the *emrA1* mutant clear subsequent *Ft* challenge very efficiently and do not exhibit extensive pathological lesions in lung, liver or spleen.

### The *emrA1* mutant vaccinated mice induce sustained production of pro-inflammatory cytokines and a potent antibody response following lethal *Ft* LVS challenge

**Fig 5:**
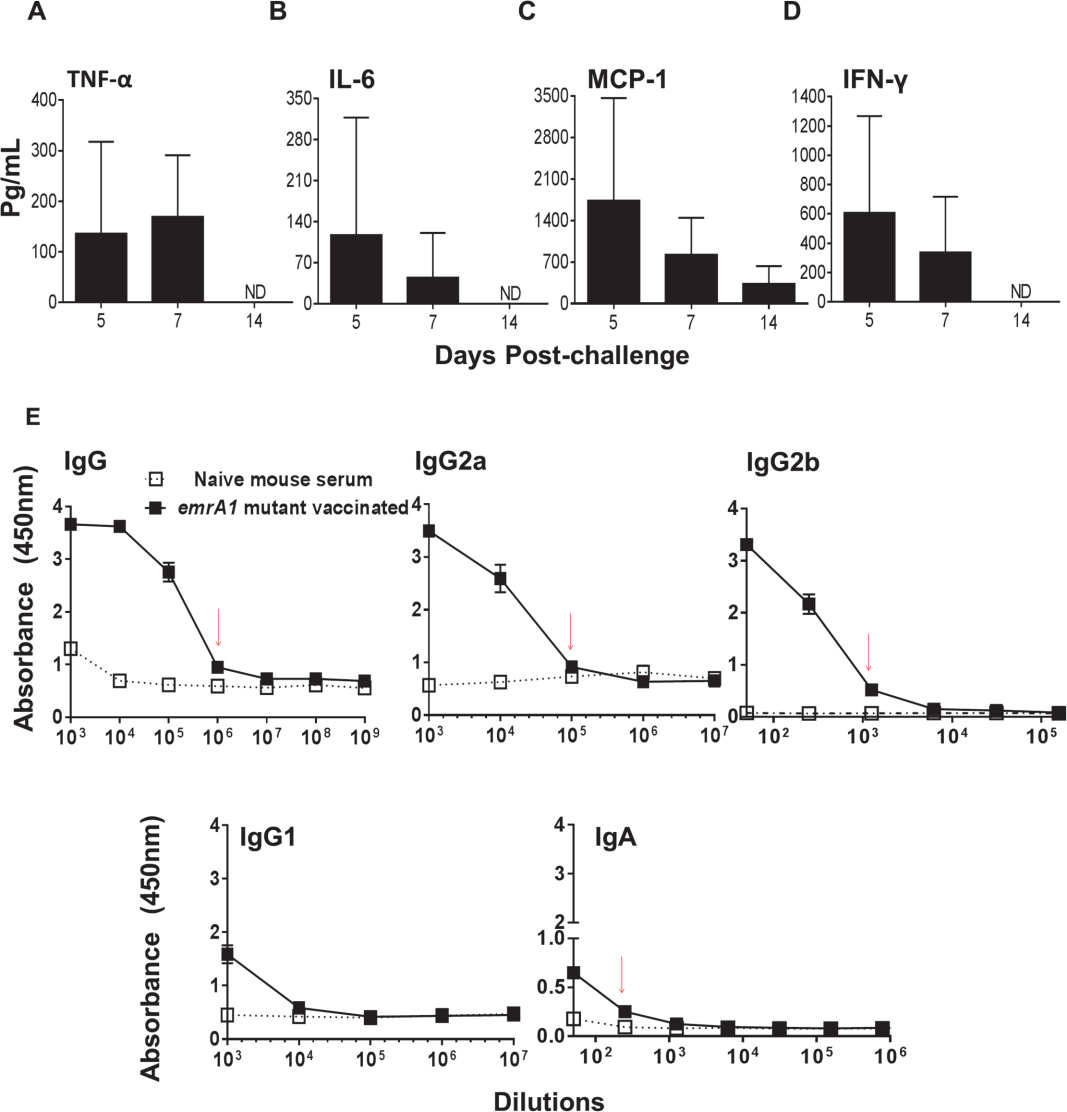
The *emrA1* mutant vaccinated mice induce sustained production of pro-inflammatory cytokines and a potent antibody response following lethal *Ft* LVS challenge. C57BL/6 mice immunized i.n. with 1×10^6^ CFU of the *emrA1* mutant were challenged i.n. with 1×10^7^ CFU of wild type *Ft* LVS 42 days post-immunization. **(A-D)** On days 5, 7 and 14 post-challenge, mice (n = 3 per group/time point) were euthanized and their excised lungs were homogenized. Clear lung homogenates were used for quantification of indicated pro-inflammatory cytokines using flow cytometric analysis. The data are represented as Mean ± S.D. **(E)** On day 21 post-challenge, mice (n = 3 per group) were anesthetized and bled retroorbitally to obtain serum. *Ft* specific total IgG, IgG2a, IgG2b, IgG1 and IgA levels in serum samples were determined by ELISA. The data are represented as Mean ± S.D. of absorbance values measured at 450 nm. Red arrows indicate antibody titers. ND = Not detected.

We next investigated the levels of pro-inflammatory cytokines in mice vaccinated with the *emrA1* mutant and challenged with 1x10^7^ CFU of *Ft* LVS on days 5, 7 and 14 post-challenge. Unlike the pro-inflammatory cytokine profiles observed following vaccination with the *emrA1* mutant, the challenged mice showed sustained production of TNF-α and MCP-1 on days 5 and 7 post-challenge. Further, the MCP-1 levels were detected until day 14 post-challenge. The IFN-γ, which was undetectable in lung homogenates of mice following vaccination with the *emrA1* mutant, was found in high levels in lung homogenates of mice challenged with *Ft* LVS on days 5 and 7 post-challenge (**Fig 5A–5D**). Collectively, these results indicate that the *emrA1* mutant vaccinated mice induce a sustained production and elevated levels of TNF-α, MCP-1 and IFN-γ following lethal *Ft* LVS challenge.

We also determined the levels of *Ft* specific total IgG, IgG2a, IgG2b, IgG1 and IgA levels in mice challenged i.n. with *Ft* LVS. It was observed that *Ft* specific total IgG, IgG2a and IgG2b antibody titers were elevated by 10–100 folds by day 21 post-challenge as compared to those observed following immunization. Additionally, similar to those observed following immunization, low levels of *Ft* specific IgG1 and IgA antibodies were observed in challenged mice (**Fig 5E)**. These results indicate that development of a potent IFN-γ-mediated innate immune response and rapid elevation of *Ft* specific total IgG, IgG2a and IgG2b levels in the *emrA1* vaccinated mice following challenge may play an important role in rapid resolution of infection.

### The *emrA1* mutant vaccinated mice are partially protected against *Ft* SchuS4 challenge

**Fig 6:**
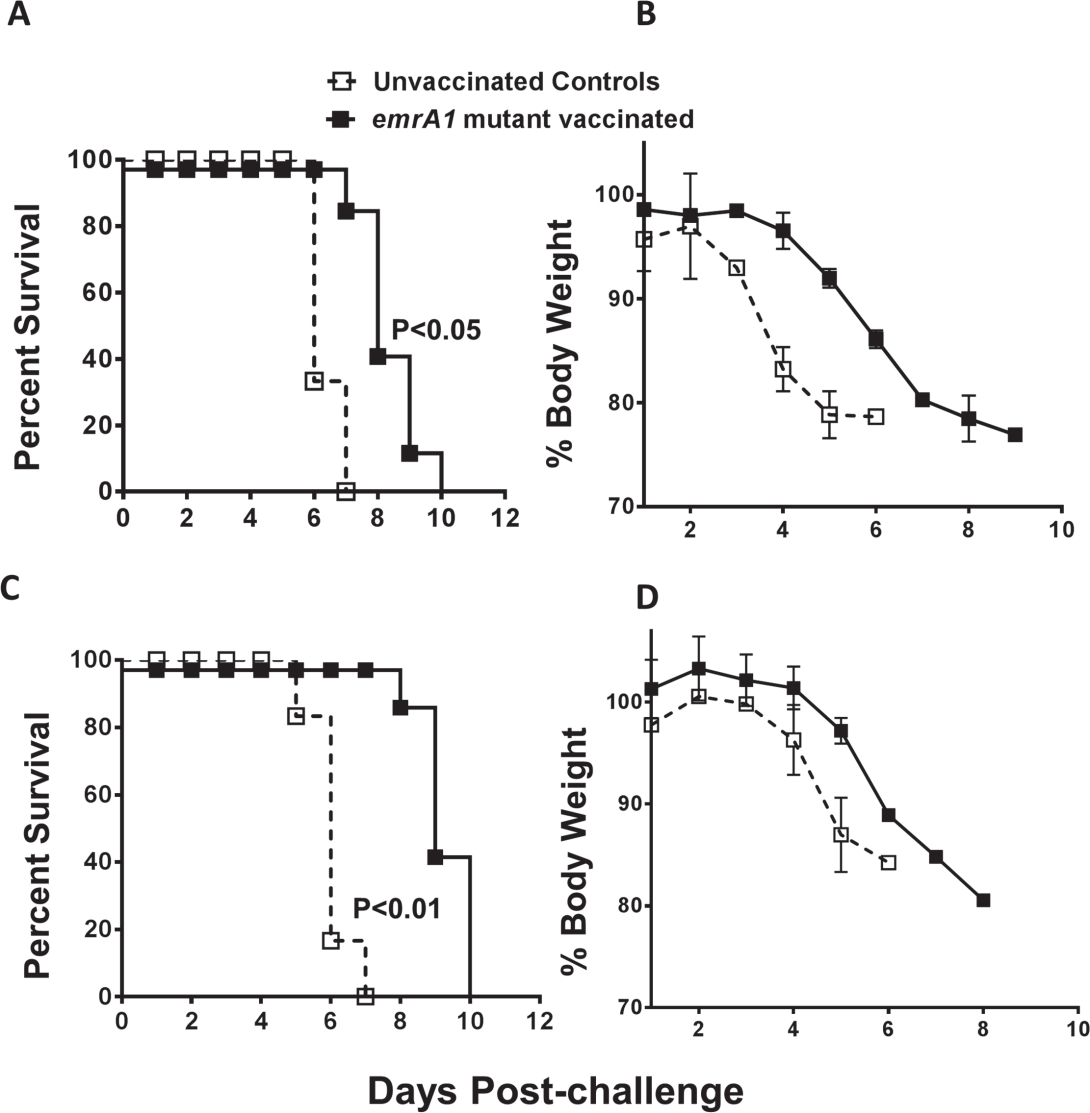
The *emrA1* mutant vaccinated mice are partially protected against *Ft* SchuS4 challenge. C57BL/6 mice (n = 10 per group) were immunized i.n. with 1×10^6^ CFU of the *emrA1* mutant. **(A)** On day 21 of the primary immunization, mice were challenged i.n. with 32 CFU of *Ft* SchuS4. Age matched unvaccinated mice challenged with a similar dose of *Ft* SchuS4 served as controls. **(B)** The mice were weighed at the indicated times post-challenge to monitor the progression of infection. **(C)** C57BL/6 mice (n = 10 per group) were immunized i.n. with 1×10^6^ CFU of the *emrA1* mutant and boosted with a similar dose on day 21. On day 42 of the primary immunization mice were challenged i.n. with 38 CFU of *Ft* SchuS4. Age matched unvaccinated mice challenged with a similar dose of *Ft* SchuS4 served as controls. **(D)** The mice were weighed at the indicated times post-challenge to monitor the progression of infection. The survival results are expressed as Kaplan-Meier survival curves and P values were determined by Log-rank test. Body weights of mice are expressed as percent body weight and represented as Mean ± S.D of percent body weight.

**Fig 7:**
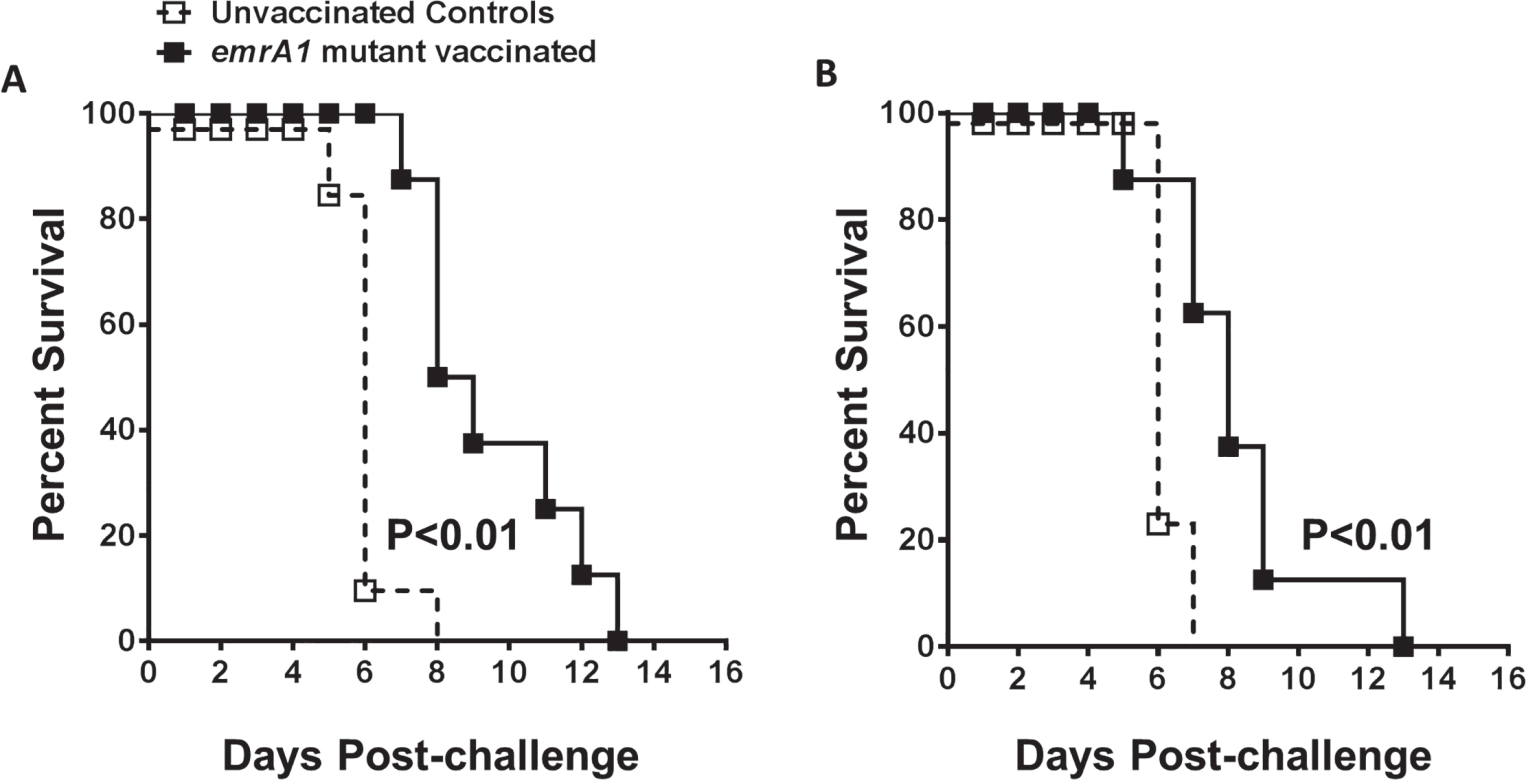
Immunization with *emrA1* mutant using a prime-boost vaccination regimen improves the extent of protection against *Ft* SchuS4 challenge. **(A)** C57BL/6 mice (n = 10 per group) were immunized i.n. with 1×10^6^ CFU of the *emrA1* mutant and boosted i.d. on day 21 with a similar dose. On day 42 of the primary immunization mice were challenged i.n. with 17 CFU of *Ft* SchuS4. Age matched unvaccinated mice challenged with a similar dose of *Ft* SchuS4 served as controls. **(B)** C57BL/6 mice (n = 10 per group) were immunized i.d. with 1×10^6^ CFU of the *emrA1* mutant and boosted i.n. on day 21 with a similar dose. On day 42 of the primary immunization mice were challenged i.n. with 17 CFU of *Ft* SchuS4. Age matched unvaccinated mice challenged with a similar dose of *Ft* SchuS4 served as controls. The survival results are expressed as Kaplan-Meier survival curves and P values were determined by Log-rank test.

Having observed a solid protection against i.n. challenge with 1000 to 10,000LD_100_ doses of *Ft* LVS, we next investigated if the *emrA1* mutant immunized mice are protected against an i.n. challenge with highly virulent and category A select agent *Ft* SchuS4. C57BL/6 mice were immunized with a single dose of 1x10^6^ CFU of the *emrA1* mutant and challenged i.n. with 32 CFU of *Ft* SchuS4. All the control mice died by day 7 with a median time to death (MTD) of 6 days. The *emrA1* mutant vaccinated mice survived longer but 100% of vaccinated mice died by day 10 with a significantly higher MTD of 8 days. The pattern of body weight loss of the control and the *emrA1* mutant vaccinated mice did not differ except that the sudden drop in weight of vaccinated mice was observed by day 6 as opposed to day 3 post-challenge observed for control mice (**Fig 6A and 6B**). In order to see if protection against SchuS4 challenge could be improved further, following a primary immunization with 1x10^6^ CFU of the *emrA1* mutant, we also gave a booster with a similar vaccination dose i.n. on day 21 of the primary immunization. The vaccinated mice were challenged with 38 CFU of *Ft* SchuS4 on day 42 of the primary immunization. However, with this vaccination regimen too, all the vaccinated mice succumbed to *Ft* SchuS4 challenge with an MTD of 9 days which was significantly higher than the control mice (MTD = 6 days) (**Fig 6C and 6D**). We further modified our vaccination strategies by immunizing a group of mice with 1x10^6^ CFU of the *emrA1* mutant i.n. followed by a booster on day 21 with a similar dose i.d. Conversely, another group was immunized first i.d. and then boosted by i.n. route. These mice were challenged with 17 CFU of *Ft* SchuS4 on day 42 of the primary immunization. All control mice died by days 6–8 post-challenge. Both the groups of vaccinated mice succumbed to *Ft* SchuS4 challenge by day 13 post-challenge with an MTD of 10 days indicating a slight improvement over the previous vaccination regimen (**Fig 7A and 7B**).

**Fig 8:**
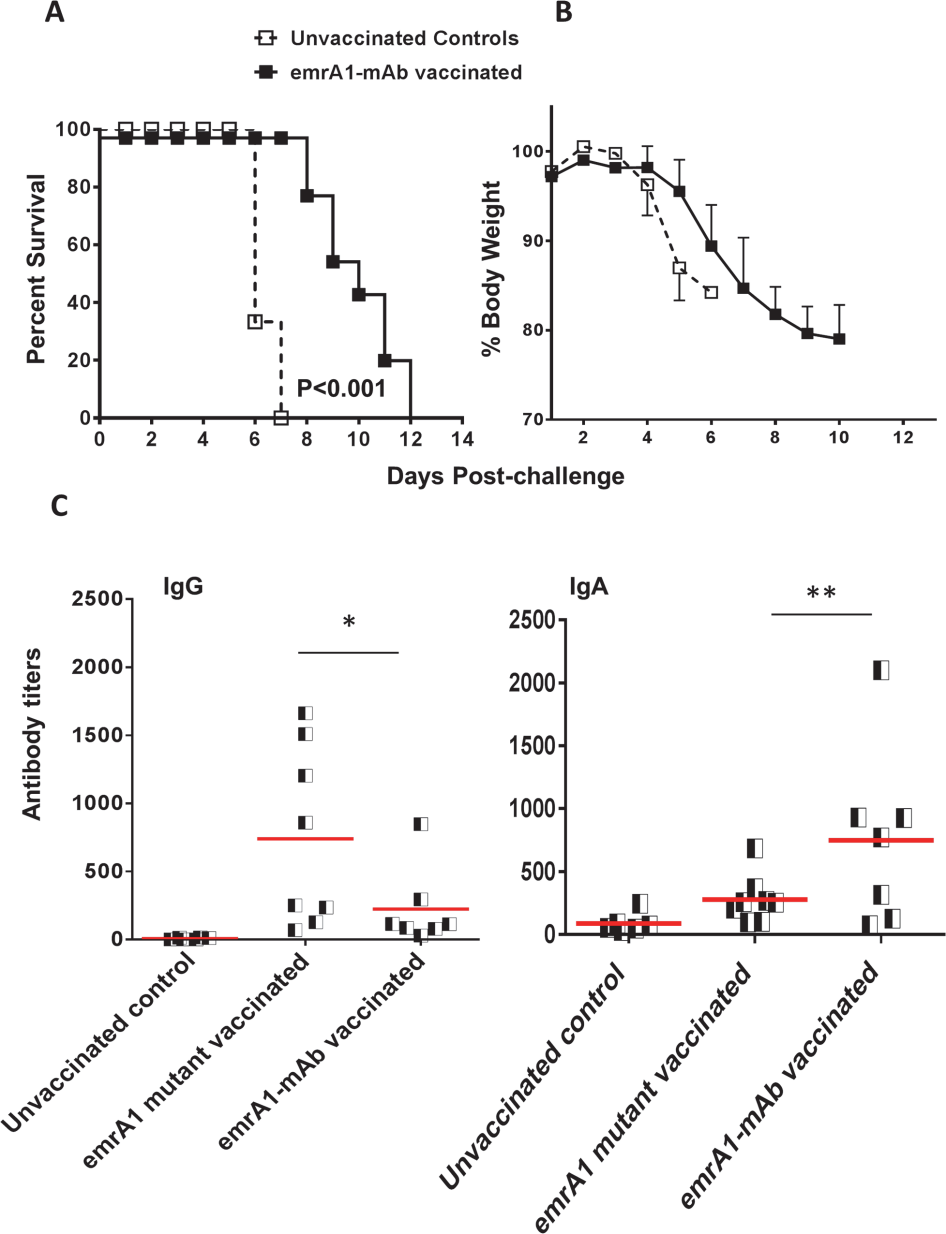
Immunization with *emrA1*-mAb complexes improves the extent of protection against *Ft* SchuS4 challenge. **(A)** C57BL/6 mice (n = 10 per group) immunized i.n. with 1×10^6^ CFU of the *emrA1* mutant-mAb immune complexes. On day 42 of the primary immunization mice were challenged i.n. with 32 CFU of *Ft* SchuS4. Age matched unvaccinated mice challenged with a similar dose of *Ft* SchuS4 served as controls. The survival results are expressed as Kaplan-Meier survival curves and P values were determined by Log-rank test. **(B)** The mice were weighed at the indicated times post-challenge to monitor the progression of infection. **(C)** The indicated *Ft* specific antibodies were determined in serum from immunized mice on day 14 post-immunization. The results are expressed as antibody titers.

**Fig 9:**
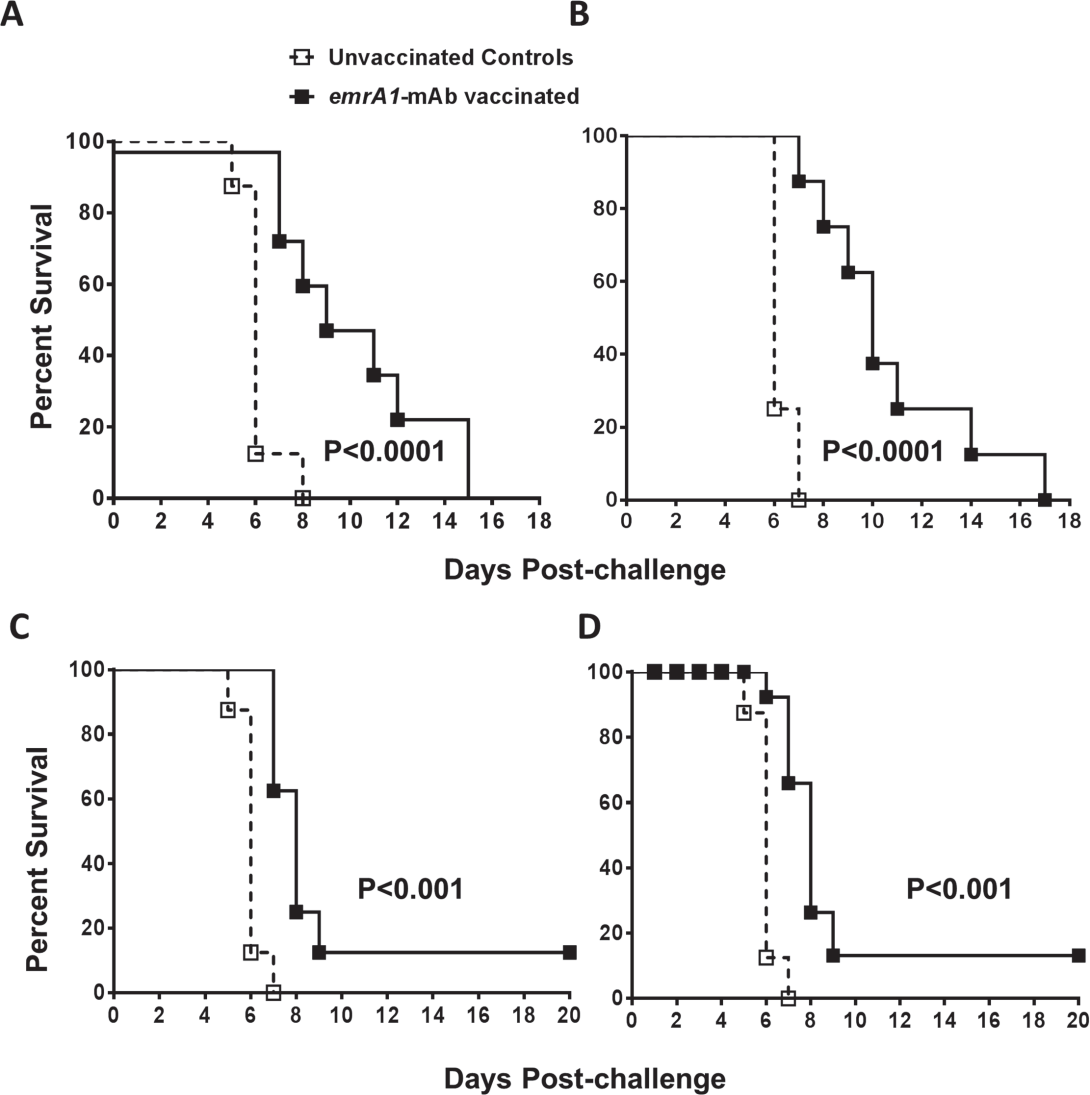
Immunization with *emrA1*-mAb complexes using a prime-boost vaccination regimen or a low dose immunization improves the extent of protection against *Ft* SchuS4 challenge. **(A)** C57BL/6 mice (n = 10 per group) were immunized i.d. with 1×10^6^ CFU of the *emrA1* mutant-mAb immune complex and boosted i.n. on day 21 with a similar dose. On day 42 of the primary immunization mice were challenged i.n. with 17 CFU of *Ft* SchuS4. Age matched unvaccinated mice challenged with a similar dose of *Ft* SchuS4 served as controls. **(B)** C57BL/6 mice (n = 10 per group) immunized i.n. with 1×10^6^ CFU of the *emrA1*mutant-mAb immune complex and boosted i.d. on day 21 with a similar dose. On day 42 of the primary immunization mice were challenged i.n. with 17 CFU of *Ft* SchuS4. Age matched unvaccinated mice challenged with a similar dose of *Ft* SchuS4 served as controls. **(C)** C57BL/6 mice (n = 10 per group) immunized i.n. with 1×10^3^ CFU of the *emrA1* mutant and boosted i.d. on day 21 with a similar dose. On day 42 of the primary immunization mice were challenged i.n. with 17 CFU of *Ft* SchuS4. Age matched unvaccinated mice challenged with a similar dose of *Ft* SchuS4 served as controls. **(D)** C57BL/6 mice (n = 10 per group) immunized i.d. with 1×10^3^ CFU of the *emrA1* mutant and boosted i.n. on day 21 with a similar dose. On day 42 of the primary immunization mice were challenged i.n. with 17 CFU of *Ft* SchuS4. Age matched unvaccinated mice challenged with a similar dose of *Ft* SchuS4 served as controls. The survival results are expressed as Kaplan-Meier survival curves and P values were determined by Log-rank test.

It has been shown that killed *Ft* LVS, when complexed with anti-*Ft* LPS monoclonal antibodies and used as a vaccine, enhances the protective efficacy against *Ft* SchuS4 challenge as compared to the killed *Ft* alone [23]. Based on this observation, we investigated next if immunizing mice with *emrA1* mutant immune complexed with the anti-*Ft* LVS LPS monoclonal antibodies improves protection against *Ft* SchuS4 challenge. A single dose i.n. immunization with 1x10^6^ CFU of the *emrA1* mutant complexed with 5μg/ml of anti-*Ft* LPS monoclonal antibodies (*emrA1-*mAb) significantly enhanced the survival of mice challenged with 32 CFU of *Ft* SchuS4. The vaccinated mice survived as long as day 12 post-challenge with an MTD significantly higher than unvaccinated controls (**Fig 8A and 8B**). We next investigated if the difference in the duration of protection offered by *emrA1*-mAb is due to differences in profile of antibody responses. Indeed, the *emrA1-*mAb mice induced significantly elevated IgA levels as early as day 14 post-immunization as compared to those immunized with the *emrA1* mutant alone (**Fig 8C**). On the contrary, mice vaccinated with the *emrA1* mutant alone induced higher levels of total IgG antibodies (**Fig 8C**). Results from experiments conducted using alternate i.n. priming and i.d. boost or vice-versa with *emrA1*-mAb allowed mice to survive as long as day 15 and day 17 post-challenge with 17 CFU of *Ft* SchuS4, respectively (**Fig 9A and 9B**). Collectively, these results demonstrate that vaccination with the *emrA1* mutant provides partial protection against *Ft* SchuS4 challenge.

Finally, we hypothesized that primary immunization and boosting with 1x10^6^ CFU of the *emrA1* mutant may result in anergy due to an antigen overload. So, we reduced the immunization dose of the *emrA1* mutant in subsequent vaccination studies. Mice were vaccinated i.n. with 1x10^3^ CFU of the *emrA1* mutant and boosted with a similar dose i.d. on day 21. Mice were challenged with 23 CFU of *Ft* SchuS4 via i.n. route. It was observed that not only mice had an extended MTD as compared to the control mice, 20% (2/10) mice survived the challenge (**Fig 9C and 9D**). These results indicate that use of alternate vaccination strategies may further improve the level of protection using *emrA1* mutant as a vaccine.

## Discussion

Several vaccination strategies have been employed for the development of an effective vaccine against tularemia since the isolation of *Ft* over a century ago. Of all the vaccination strategies, use of live *Ft* LVS or the mutants generated on *Ft* LVS, *Ft* SchuS4, or *F*. *novicida* backgrounds have been successful in providing protection against respiratory challenge with *Ft* SchuS4 [24,25,14,26,27,28]. Studies have shown that both antibodies and T-cell responses are required for effective protection against virulent *Ft* strains [29]. This explained why killed or subunit vaccines failed to provide adequate protection against *Ft* SchuS4. The *Ft* LVS was developed in the 1950s by multiple passages of the *Ft holarctica* (type B strain) on peptone cysteine agar. These passages led to spontaneous mutations in the genome that made it relatively avirulent to humans while retaining its virulence in mice [30]. However, due to the lack of genetic analysis tools at the time, the exact nature of these spontaneous mutation(s) could not be determined. Modern sequencing technology has revealed that repeat-mediated deletions occurred in two genes namely *pilA* encoding a putative type IV pilin and FTT0918 encoding a putative outer membrane protein [31]. These mutations collectively led to the attenuation seen in *Ft* LVS. Reintroduction of these deleted foci into *Ft* LVS restores virulence back to levels of parent type B strain [32]. *Ft* LVS, although attenuated for virulence in humans has yet to be approved as a vaccine for mass immunization in the USA due to various reasons such as possibility of reversion, as well as cases of vaccine induced tularemia [33]. Furthermore, it confers poor immunity against high dose aerosol challenges with the virulent SchuS4 strain [34] and possesses potent immunosuppressive properties [35,22]. Currently, *Ft* LVS is used only for immunization of personnel at a high risk of infection [36]. Thus a vaccine generated on *Ft* LVS background lacking the traits that undermine its vaccine potential may prove useful in prevention of tularemia.

EmrA1 is a transmembrane component of the multidrug efflux pumps belonging to Major Facilitator Superfamily (MFS) of transporters. We have reported that the *emrA1* mutant of *Ft* LVS is sensitive to several oxidants and antimicrobial agents. The *emrA1* mutant exhibits diminished intramacrophage growth that can be restored to the wild type *Ft* LVS levels either by inhibition of reactive oxygen species by chemical inhibitors or infection in NADPH oxidase deficient macrophages [20]. We have further shown that oxidant sensitivity of the *emrA1* mutant is due to the failure to secrete antioxidant enzymes SodB and KatG. Further characterization of the *emrA1* mutant revealed that it exhibits oxidant sensitive phenotypes of both the *sodB* and Δ*katG* mutants [20]. Based on these properties of the *emrA1* mutant, we hypothesized that the *emrA1* mutant will provide superior protection against respiratory tularemia caused by *Ft*. We tested the vaccine potential of the *emrA1* mutant in prevention of respiratory tularemia caused by *Ft* in this study.

Attenuated mutants of *Ft* LVS when used as vaccines have been shown to offer protection in mice against i.d. challenge with *Ft* SchuS4 strain [37,38,25]. Similarly, attenuated mutants of Type A *Ft* strains when used as vaccines protect against a homologous SchuS4 challenge [28,39] [40] [41]. A low dose immunization with the attenuated mutants of SchuS4 either by i.n. or i.d. routes have been shown to protect mice from a low dose (10 CFU) i.n. challenge. Contrarily, a high dose i.d. immunization with these mutants do not protect immunized mice against a higher (200 CFU) i.n. challenge dose [14]. In majority of these studies, protection against i.n. challenge with *F*. *tularensis* SchuS4 strain was achieved only in BALB/c mice and the mutants used in these studies were either not tested or failed to provide protection in C57BL/6 mice. Furthermore, these studies did not use prime-boost vaccination strategies probably due to the residual virulence of the mutants and/or potential for adverse effects due to repeated immunizations. The results from our present study demonstrate that *emrA1* mutant is sufficiently attenuated and repeated high dose booster vaccinations can be administered to improve the level of protection against i.n. challenge with *Ft* SchuS4 strain without causing any adverse reaction in the immunized mice.

Our results show that *emrA1* mutant is safe and does not cause any adverse effects in mice even when administered at a dose as high as 1x10^6^ CFU by i.n. route. Mice exhibited weight loss for a brief period but regained it quickly, showed minimal-to-none histopathological lesions in lung, liver and spleen and cleared the bacteria by day 14 post-immunization. The immunized mice were solidly protected against an i.n. challenge dose as high as 1000 to 10,000 LD_100_ of *Ft* LVS. Such a high degree of protection has not been reported earlier against respiratory challenge with *Ft* LVS using a single immunization dose with attenuated mutant generated on *Ft* LVS background. A single immunization with lower doses (1x10^4^ and 1x10^5^ CFU i.n.) of *emrA1* mutant reduced the efficacy of protection against 1x10^7^ CFU challenge dose but did provide protection against lower challenge doses (not shown). Further, the fact that challenged mice clear the bacteria efficiently without exhibiting a significant weight loss or histopathological lesions are indicative of robust protective immunity. Additionally, 100% protection observed in mice challenged with 1x10^7^ CFU of *Ft* LVS after 75 days of a single dose immunization indicates that *emrA1* vaccination induces a long-lasting immunity.

The correlates of immune protection that can provide various quantifiable markers for a successful vaccination have not been very well established for tularemia [42,43]. Although antibodies are required for protection, they have been judged as a poor correlate of immune protection. However, levels of TNF-α, IFN-γ, MCP-1 and IL-17 that are upregulated on activation of T cells have been shown to be the markers of a successful vaccination [44,21]. A sustained pro-inflammatory cytokine response consisting of high levels of TNF-α, IFN-γ and MCP-1 in the *emrA1* mutant vaccinated mice following i.n challenge with *Ft* LVS is indicative of a strong recall response. An enhancement of antibody mediated immune response immediately after challenge also indicates that a concerted action of both the humoral and cell mediated arms of adaptive immune response causes rapid resolution of infection. However, despite the solid protection observed against high challenge doses of *Ft* LVS, the *emrA1* mutant vaccinated mice were only partially protected against the virulent *Ft* SchuS4 challenge. Single and prime boost strategies using alternate i.n. and i.d. vaccination schedules significantly increased the MTD against *Ft* SchuS4 challenge, but eventually all the vaccinated mice succumbed to infection. Surprisingly, priming and boosting with a low dose of *emrA1* mutant not only increased MTD but also protected 20% of the vaccinated mice against lethal *Ft* SchuS4 challenge. Moreover, when these surviving mice were sacrificed and their lung homogenates plated at the termination of the experiment, they were found to carry *Ft* SchuS4 in lungs indicating that these mice did receive the challenge dose and were able to keep the infection under control. These results are intriguing and are being investigated further.

The unexpected lack of protection observed in the *emrA1* mutant vaccinated mice against *Ft* SchuS4 is surprising and reaffirms the previous observations that immune components required for protection against *Ft* LVS differ from those required for *Ft* SchuS4. The choice of C57BL/6 mouse model in vaccination studies to demonstrate protection against *Ft* SchuS4 challenge is based on the assumption that any vaccine candidate that can protect this hard-to-protect model will be an indicator of its protective efficacy in humans. However, this assumption is proven wrong by a recent study. It has been reported that *iglD* mutant of *F*. *novicida* fails to provide any protection in mice against a pulmonary challenge with *Ft* SchuS4 [45]. However, this mutant when used as a vaccine protected Fisher 344 rats and non-human primates against *Ft* SchuS4 challenge. Additionally, in this study when *Ft* LVS was compared in parallel with *iglD* mutant, it provided protection in both these animal models against *Ft* SchuS4 challenge. It is significant to mention that similar to *iglD* mutant, immunization with *Ft* LVS does not protect C57BL/6 mice against *Ft* SchuS4 challenge [45]. Thus, based on these observations, we speculate that the *emrA1* mutant when used as a vaccine in Fisher rats or non-human primate models may render 100% protection against *Ft* SchuS4 challenge. Future studies are directed at testing the vaccine potential of the *emrA1* mutant in these two animal models.

Our previous studies have shown that a mutant of *Ft* LVS deficient in iron-containing superoxide dismutase B (SodB) is superior to *Ft* LVS in protecting C57BL/6 mice against a respiratory *Ft* SchuS4 challenge [13]. We demonstrated that superior protection offered by the *sodB* mutant is due to its attenuated virulence as compared to wild type *Ft* LVS and upregulation of several immunogenic proteins resulting from oxidative stress [13]. Further, we have demonstrated that the antioxidant enzyme KatG of *Ft* LVS is involved in the suppression of host’s innate immune response by inhibiting the redox-sensitive signaling components [18]. Moreover, a mutant of *Mycobacterium bovis* BCG in iron-containing superoxide dismutase (SodA) strain has shown to confer better protection in immunized mice than the parent BCG strain against virulent *M*. *tuberculosis* challenge [19]. A modified BCG containing a mutation in *sodA* gene and sigma factor SigH which regulates the expression of several genes required for resistance to oxidative stress, induced stronger immune responses than the parent BCG during primary vaccination as well as an enhanced recall response upon subsequent challenges resulting in superior protection against *M*. *tuberculosis* challenge [46,47]. These observations indicate that antioxidant enzymes may be targeted to develop effective vaccines against bacterial pathogens. In the context of tularemia prevention, antioxidant defenses of *Ft* may specifically be targeted to improve the protective efficacy and reduce the immunosuppressive properties of parent *Ft* LVS strain. Moreover, we speculate that given a 100% identity of nucleotide and amino acid sequences of the *emrA1* gene and protein between *Ft* LVS and SchuS4, the *emrA1*mutant of *Ft* SchuS4 may have a phenotype similar to that observed for the *emrA1* mutant of *Ft* LVS. However, a more rigorous assessment of the vaccine potential would require generation of *emrA1* mutant in the *Ft* SchuS4 background. To conclude, results from the present study indicate that *Ft* antioxidant mutants may be developed into effective vaccines for tularemia prophylaxis.
